# Myeloperoxidase as an Active Disease Biomarker: Recent Biochemical and Pathological Perspectives

**DOI:** 10.3390/medsci6020033

**Published:** 2018-04-18

**Authors:** Amjad A. Khan, Mohammed A. Alsahli, Arshad H. Rahmani

**Affiliations:** Department of Medical Laboratories, College of Applied Medical Sciences, Qassim University, AlQassim, P.O. Box 6699, Buraidah 51452, Saudi Arabia; shly@qu.edu.sa (M.A.A.); ah.rahmani@qu.edu.sa (A.H.R.)

**Keywords:** myeloperoxidase, leukocytes, inflammation, oxidative stress, chronic diseases, disease biomarker

## Abstract

Myeloperoxidase (MPO) belongs to the family of heme-containing peroxidases, produced mostly from polymorphonuclear neutrophils. The active enzyme (150 kDa) is the product of the *MPO* gene located on long arm of chromosome 17. The primary gene product undergoes several modifications, such as the removal of introns and signal peptides, and leads to the formation of enzymatically inactive glycosylated apoproMPO which complexes with chaperons, producing inactive proMPO by the insertion of a heme moiety. The active enzyme is a homodimer of heavy and light chain protomers. This enzyme is released into the extracellular fluid after oxidative stress and different inflammatory responses. Myeloperoxidase is the only type of peroxidase that uses H_2_O_2_ to oxidize several halides and pseudohalides to form different hypohalous acids. So, the antibacterial activities of MPO involve the production of reactive oxygen and reactive nitrogen species. Controlled MPO release at the site of infection is of prime importance for its efficient activities. Any uncontrolled degranulation exaggerates the inflammation and can also lead to tissue damage even in absence of inflammation. Several types of tissue injuries and the pathogenesis of several other major chronic diseases such as rheumatoid arthritis, cardiovascular diseases, liver diseases, diabetes, and cancer have been reported to be linked with MPO-derived oxidants. Thus, the enhanced level of MPO activity is one of the best diagnostic tools of inflammatory and oxidative stress biomarkers among these commonly-occurring diseases.

## 1. Introduction

Myeloperoxidase (MPO) (EC 1.11.1.7) is a member of subfamily of peroxidases. It is most abundantly expressed in immune cells, such as neutrophilic polymorphonuclear leukocytes (neutrophils) and lymphocytes [1,2], monocytes, and macrophages [3], and is also produced in other body cells. Myeloperoxidase is stored in cytoplasmic membrane-bound azurophilic granules and, during stimulation, these granules are secreted out to the extracellular space by degranulation or exocytosis [4,5]. The complete biochemical mechanism of neutrophil degranulation is not yet clear, but oxidative stress plays a key role in the release of MPO from these cells [6,7]. 

Neutrophils are well known white blood cells (WBCs) playing a pivotal role in innate immunity and frontline defense against microbial attacks [8]. In addition to MPO, several other proteins or enzymes are present in neutrophils which also show antimicrobial properties, e.g., defensins, serine proteases, cathepsin G, alkaline phosphatase, lysozyme, NADPH oxidase, collagenase, lactoferrin, cathepsin, and gelatinase, etc. [9]. Among these antimicrobial agents, MPO is the most abundant and constitutes 5% dry weight of neutrophils and 25% of the azurophilic granular proteins [10].

Normally, neutrophils degranulate at the infection site to combat different types of microbial activities and help to cure diseases. However, any unusual expression and release of MPO from activated neutrophils intensifies the inflammation and tissue damage and may result in several other diseases, even in the absence of infection [8,11]. 

This review article mainly focuses on the recent advances in the biochemical and the pathological aspects of myeloperoxidase and its significance as a disease biomarker in some commonly occurring chronic diseases.

## 2. Biochemistry of Myeloperoxidase

**Figure 1:**
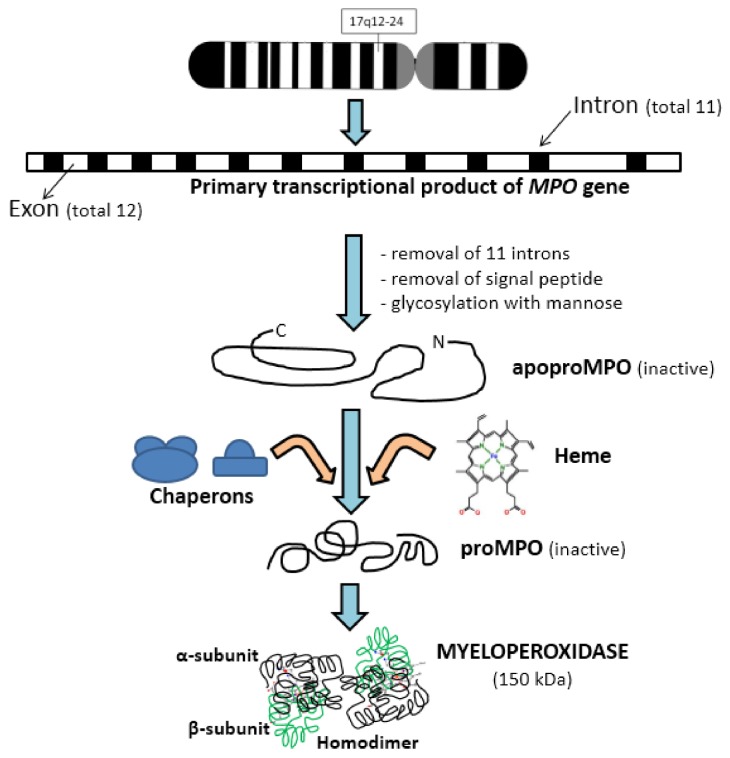
Sequential steps involved in the synthesis of myeloperoxidase. MPO: myeloperoxidase.

The *MPO* gene is located on the long arm segment q12–24 of chromosome 17 and the primary transcriptional product of this gene consists of 11 introns and 12 exons [12,13]. After some modifications like signal peptide removal and glycosylation with mannose-rich side chains, it produces apoproMPO [14]. This protein product is enzymatically inactive and further forms complexes with some chaperons like calreticulin and calnexin in the endoplasmic reticulum [15,16]. Enzymatically inactive, proMPO is formed from apoproMPO by the insertion of a heme moiety [17]. Furthermore, the removal of some N-terminal amino acids results in the production of 72–75 kDa protein, which undergoes further cleavage to produce α and β subunits. The α-subunit is heavy, 57 kDa, and consists of 467 amino acids, while the β-subunit is light, 12 kDa, and consists of 112 amino acids (Figure 1).

Mature MPO consists of cationic homodimer heavy-light chain protomers and is about 150 kDa by weight. Each heavy subunit of mature MPO is covalently linked with a heme group and a mannose-rich moiety [18,19]. On the basis of the size of heavy chains in MPO, three isoforms have been observed: MPO I, MPO II, and MPO III [20]. A calcium-binding site is also present in MPO, which is very important for active site structure and function [21]. 

### 2.1. Activation and Release of Myeloperoxidase by Neutrophils

Although the coordination of MPO release by the degranulation of neutrophils is not fully understood, evidence supports that increased levels of oxidative stress by reactive oxygen species (ROS) and the activation of Src and p38 mitogen activated protein (MAP) kinase signaling pathways performs prominent roles in the process [22,23]. 

A fine coordination is necessary between different biochemical pathways, such as neutrophil activation, the production of ROS by superoxide generating NADPH oxidase, and MPO release by exocytosis. All of these organized reactions lead to the elimination of the bacterial invasion. Invading bacteria initiate enhanced production of H_2_O_2_ by superoxide dismutase (SOD), which is utilized by MPO for the production of chloramine and hypochlorite. Both of these products are highly toxic for the invading bacteria [8,24]. This biochemical phenomenon is also called respiratory burst. A clear illustration of the role of MPO is also observed in MPO knockout mice, which are highly susceptible to infections by *Klebsiella* and *Candida* and show persistent inflammation [25,26].

During bacterial infection, various other pro-inflammatory factors also trigger the release of MPO and ROS from neutrophils. During bacterial infection, one of the important mediators for this cascade is formylated peptide, which also works as a chemoattractant. Neutrophils are activated by this chemoattractant via formyl peptide receptor (fPR), a G protein-coupled receptor [27]. Some more proteins involved in antibacterial activities include phospholipases and protein kinases, such as mitogen-activated protein kinases (MAPK) and protein kinase C (PKC) [28,29,30]. During different pathological situations, or by the influence of several drugs, this signaling cascade gets impaired and finally leads to neutrophil dysfunction. These aberrations can be detrimental to host defense against several diseases or disease-causing microorganisms [27,31,32]. 

### 2.2. Reaction Mechanism and Functions of Myeloperoxidase

Activated neutrophils, monocytes, and some tissue macrophages release MPO at the sites of inflammation, using H_2_O_2_ to oxidize several substrates, such as halides (Cl^−^, Br^−^, and pseudohalides like thiocyanate (SCN^−^). This reaction leads to the formation of hypohalous acid, hypochlorous acid (HOCl^−^), hypobromous acid (HOBr^−^), and hypothiocyanous acid (HOSCN) [33]. MPO is able to interact with diverse ionic, atomic, and molecular entities via the interface with H_2_O_2_, including HOCl^−^, hydroxyl radicals, singlet oxygen, ozone, chloramines, and aldehydes [34,35,36]. These species are potent oxidants, which under normal and controlled circumstances are toxic to several microorganisms and play an important role in the immune system [37,38,39]. However, any excessive or unregulated production of theses oxidants can lead to damage of host cells and result in several diseases.

**Figure 2:**
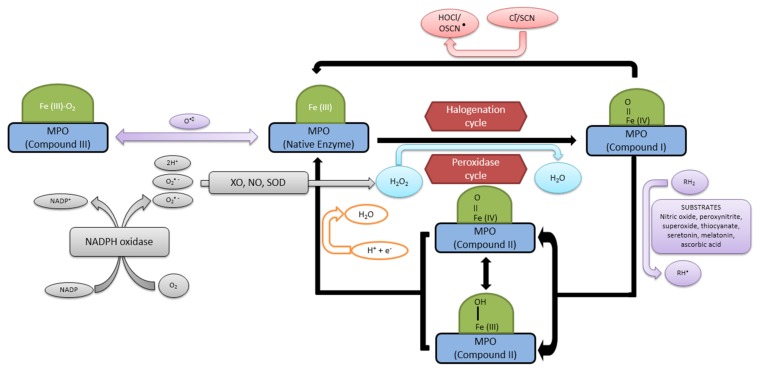
Reaction mechanism of myeloperoxidase in the presence of different substrates. XO: xanthine oxidase; NO: nitric oxide; SOD: superoxide dismutase; Cl^−^/SCN: chloride/thiocyanate.

Myeloperoxidase produces reactive oxidants and other free radicals either through its peroxidase cycle or through a halogenation cycle, depending up on the substrate availability [40]. It is the only type of peroxidase that facilitates the oxidation of chloride to HOCl^−^ by consuming H_2_O_2_. During its reaction cycles, MPO is converted to many transitional forms with different half-lives. Myeloperoxidase contains ferric heme (MPO-Fe(III)) in its resting state. During the peroxidase cycle, compound I is formed by the reaction with H_2_O_2_ [41]. In the absence of Cl^−^, the intermediate (MPO-Fe(IV)=O) in the presence of peroxide is reduced back to the ferric state in two sequential steps. The first step leads to the formation of compound II. This compound is reduced to compound III by second equivalent AH_2_ [42,43]. During the halogenation cycle, MPO-compound I exclusively oxidizes Cl^−^ to HOCl^−^ and no further intermediates are formed in this reaction, as compound I gets converted directly to its native form (Figure 2). 

The fate of H_2_O_2_ as a substrate of MPO via peroxidation or chlorination reaction depends upon the concentration of chloride and the reducing substrates. A number of sources such as NADPH oxidase, xanthine oxidase, and nitric oxide synthase (NOS) are the sources of H_2_O_2_ for the MPO reaction, which also enhance the oxidative potential of H_2_O_2_ [44].

A strong antimicrobial cascade of reactions (respiratory burst), takes place in the presence of NADPH oxidase [45]. The initial products of this reaction are superoxide anions (O_2_^−^), produced as NADPH + O_2_ → O_2_^−^ + NADP^+^ + H^+^.

During normal conditions, the antibacterial activities of MPO involve the production of different reactive oxygen and nitrogen species (ROS and RNS, respectively). These ROS and RNS also cause lipid peroxidation, protein nitration, and protein carbomylations. Myeloperoxidase plays a role in the oxidation and chemical modifications of different lipoproteins as well. In addition to this, MPO also mediates protein nitrosylation, dityrosine crosslinking, and 3-chlorotyrosine formation [46,47].

With the help of H_2_O_2_, this enzyme also oxidizes tyrosine to the tyrosyl radical. Neutrophils use HOCl^−^ and the MPO-derived tyrosyl radical as cytotoxic agents against different types of bacteria and other pathogens [48]. As a signaling molecule, HOCl can activate several pathways, which later induce cellular senescence or apoptosis [49].

The polycationic protein nature of MPO helps it to bind several negatively charged surfaces of pathogens and causes cell membrane destruction, which ultimately leads to cell death [50]. In addition to pathogens, this enzyme can bind other cell surfaces like epithelial cells, fibroblasts, endothelial cells, macrophages, platelets, neutrophils, and low-density lipoproteins (LDLs) and very low-density lipoproteins (VLDLs) [51,52,53,54,55,56,57,58,59]. The binding of this enzyme to these cell surface alters some functional properties; for example, interaction with neutrophil integrins causes enhanced tyrosine phosphorylation of some proteins. This activates protein tyrosine kinase, which results in degranulation and leads to respiratory burst [58]. The binding of MPO to platelets causes the reorganization of the platelet cytoskeleton, and thus alters the aggregation properties [60]. MPO also oxidizes a variety of aromatic compounds by a one-electron mechanism to produce substrate radicals (R**˙**) [61,62].

In contrast to activation, different types of cytoplasmic enzymes are indirectly inactivated by myeloperoxidase-derived HOCl, due to its high chemical reactivity. Some of these enzymes include creatine kinase, lactate dehydrogenase, hexokinase, glyceraldehyde-3-phosphate dehydrogenase, etc. [63].

In addition to the many positive roles of myeloperoxidase discussed above, this enzyme has some drawbacks as well. Carbon nanotubes are used as drug delivery vehicles by some clinicians, but myeloperoxidase remains a significant hurdle as this enzyme suddenly breaks down these vehicles, thus limiting its applications [64].

### 2.3. Measurement of Myeloperoxidase Activity

Myeloperoxidase assays have been widely reported in the literature. However, no proper unanimity for most standard assays has been established. The complications are due to the fact that the substrates of MPO are the same as general peroxidase substrates. In addition to this, myoglobin and hemoglobin also show some peroxidase activity, thus interfering with the actual results. Unfortunately, no comparisons have been made between different myeloperoxidase assays, so standardization and validation are the first priorities in confirming the results from various studies [65]. Myeloperoxidase can be detected by flow cytometry, immunohistochemistry, or cytochemical staining. Some of the common assays of MPO are briefly described below. 

Myeloperoxidase assays have been commonly performed by using different substrates, such as tetramethylbenzidine (TMB), 10-acetyl-3,7-dihydroxyphenoxazine (ADHP), and *o*-dianisidine dihydrochloride. These substrates react in the presence of H_2_O_2_ and form different colored products, which are estimated colorimetrically [66,67,68].

Myeloperoxidase assays have also been checked through chlorination activity evaluated with 39-(*p*-aminophenyl) fluorescein (APF) and 39-(*p*-hydroxyphenyl) fluorescein in the presence of H_2_O_2_. The fluorescence by this assay is checked by spectrofluorometry [65]. Myeloperoxidase activity has also been assayed by bromide-dependent chemoluminescence using luminol in the presence of H_2_O_2_ [69]. 

Nowadays, the most common method of MPO measurement is via commercially-available enzyme-linked immunosorbent assay (ELISA) kits. The MPO level is measured by sandwich ELISA with a monoclonal antibody [70]. Human plasma diluted samples, a control, and a standard are pipetted in wells coated with biotin-conjugated mouse anti-human MPO monoclonal antibodies bound to streptavidin–horseradish peroxidase (HRP) and incubated on a vibrating shaker at room temperature. After some time, the contents of the wells are washed and replaced with TMB as a substrate, and then incubated for a specific time. This reaction mixture is followed by a stop solution. Absorbance is determined by an ELISA reader and the quantity of MPO is interpreted from the calibration curve of the standard. The assay is sensitive (the lower limit of detection may be 0.026 ng/mL) [71].

### 2.4. Inhibitors and Activators of Myeloperoxidase

Even though a strong correlation has been found between atherosclerosis, inflammatory diseases, and MPO release, little work has been done to inhibit MPO to suppress these diseases. Several naturally occurring compounds possess inhibitory activities against MPO, including polyphenols, melatonin, flavonoids, etc. [72]. 

The MPO reaction is inhibited by general peroxidase inhibitors azide and benzoic acid hydrazide-containing compounds, but the proper mechanism of its inhibition is still unknown [73]. There are some specific inhibitors of MPO, such as 4-amino benzoic acid hydrazide (4-ABH) [74]. Ceruloplasmin, an acute phase plasma protein produced from hepatocytes and activated macrophages, is a physiologic inhibitor of MPO [75]. Niacin can also inhibit cellular ROS production and MPO release through some complex signaling mechanisms [76].

In addition to the above compounds, some naturally occurring anti-inflammatory, antioxidant, and antihistaminic compounds possess inhibitory activities against MPO. These compounds include nonsteroidal anti-inflammatory drugs (NSAIDs), e.g., diclofenac, ferulic acid, caffeic acid, resveratrol, indomethacin, flufenamic acid, chalcones, and gallic acid [77,78,79].

As compared to inhibitors, little is known about the activators of MPO. This type of peroxidase is present as an inactive or partially active form in resting granulocytes and its activation is instigated by different factors. Some of the activators discovered include granulocyte macrophage colony stimulating factor (GM-CSF), *N*-formyl-methionyl-leucyl-phenylalanine (fMLP), and phorbol mysristate acetate [80,81].

## 3. Role of Myeloperoxidase in Different Diseases

In addition to the antipathogenic or bactericidal role of MPO-derived HOCl during normal conditions, under some pathological circumstances the overproduction of these oxidizing agents also causes oxidative damage of proteins and DNA in host cells. Several types of tissue injuries and the pathogenesis of various chronic diseases such as atherosclerosis, cancer, renal disease, lung injury, and multiple sclerosis. Additionally, Alzheimer’s and Parkinson’s diseases have been reported to be directly/indirectly linked with MPO-derived oxidants [8] (Table 1). Thus, the enhanced level of MPO is one of the best inflammatory and oxidative stress markers among these commonly occurring diseases [82,83]. 

### 3.1. Inflammation

Some of the common examples of diseases and conditions with chronic inflammation are: tuberculosis, asthma, rheumatoid arthritis, chronic sinusitis, chronic hepatitis, peptic ulcer, ulcerative colitis, and chronic periodontitis. Myeloperoxidase is now considered as a new biomarker of inflammation in these diseases as well as other ailments like ischemic heart disease and acute coronary syndrome. This enzyme is released into the extracellular medium during different inflammatory processes [111]. During inflammation, vascular permeability is increased by the activation of various inflammatory mediators, which results in the influx of immunoglobulins and serum proteins at the site of inflammation [112,113]. This cascade of inflammatory process also motivates the migration of polymorphonuclear neutrophils, which result in the release of MPO [114,115]. 

Inflammatory processes are also triggered by lipid peroxidation and the synthesis of eicosanoids. Cytochrome P_450_, lipoxygenase, and cyclooxygenase also play a prominent role in these events. Myeloperoxidase generates reactive intermediates that stimulate lipid peroxidation. This oxidoreductase can oxidize tyrosine and nitrite to form tyrosyl radical and nitrogen dioxide (**˙**NO_2_), respectively. These reactive intermediates can oxidize lipids in plasma and the cell membrane [116]. The lipoprotein phospholipid peroxidation of the membrane is linked to their interference, leading to cellular dysfunctions. Lipid peroxidation can be a normal physiological activity or a potential contributor to the pathophysiological consequence of acute and chronic inflammatory diseases [117,118].

Myeloperoxidase also leads to the formation of tyrosyl radical, formed when MPO initiates lipid peroxidation, also leading to the formation of phenolic cross-links on proteins. A typical molecular fingerprint, protein-bound dityrosine, is enhanced during atheroma and other sites of inflammation [119]. In addition to this, lipid peroxidation also occurs by nitrogen dioxide (**˙**NO_2_), which is formed by MPO enzymatic action. Myeloperoxidase is also indirectly involved in the post-translational modification of some proteins, resulting in the formation of nitrotyrosine, which can also occur in the presence of **˙**NO_2_ [120,121].

### 3.2. Rheumatoid Arthritis

Elevated MPO levels have been observed in several inflammatory diseases including rheumatoid arthritis (RA) [122]. Rheumatoid arthritis is well characterized by chronically inflamed synovial joints with some destruction of cartilage and bones [123]. Several factors have been proposed for this disease, among which oxidative stress is a leading hypothesis [124,125]. Inflamed synovium is often intervened by B and T lymphocytes, macrophages, and neutrophils. The intrusion of these cells in the synovium during RA leads to the release of multiple pro-inflammatory mediators. The degranulation of neutrophils leads to the release of enzymes and peptides, leading to respiratory burst and oxidative stress [126,127,128,129]. The overproduction of ROS is a potential tissue-damaging agent that is further formed by the cascades of reactions by HOCl^−^ produced by the activated neutrophils present in synovial fluid [101,130]. This was verified in the inflamed cartilage of patients suffering from RA [102]. Currently, a firm hypothesis is that the enhanced levels of MPO in inflamed cartilage of RA are causally associated with the lifelong disease progression.

### 3.3. Cardiovascular Diseases and Atherosclerosis

Myeloperoxidase gained special importance in 2001, due to its association with coronary artery diseases (CAD). Since that time, MPO has been considered a circulating marker of related diseases such as acute coronary syndrome, CAD, and chronic heart failure [131,132,133,134,135]. Elevated levels of circulating MPO are observed in patients with coronary artery diseases, unstable angina, and acute myocardial infarction [8,136,137,138]. Plasma MPO concentration was reported to be higher in myocardial infarction (MI) patients (55 ng/mL) as compared to control subjects (39 mg/mL) [139]. 

Circulating MPO also shows a link with red blood cells (RBCs)’ rigidity index in several patients with combined ischemic heart disease. This enzyme induces some changes in RBCs’ cellular morphology and biophysical properties like plasma membrane fluidity, transmembrane potential, cell size, hemolysis sensitivity, and cellular deformability. This enzyme also alters the intracellular Ca^2+^ level in addition to causing some changes in band 3 proteins and glycophorins of the RBC membrane [60].

Atherosclerosis is the major cause of cardiovascular diseases (CVD). Neutrophils and monocytes play a key role in atherosclerosis, leading to chronic inflammatory problems. Different events and sequences occur during CVD, which include endothelial dysfunction besides the formation and rupture of atherosclerotic plaque [140]. In the arterial wall subendothelial region, all of these stages occur during inflammation, which ultimately leads to the accumulation and deposition of altered lipids [141].

Atherosclerosis leads to the accumulation of cholesterol and cholesteryl esters on arterial walls, which are derived from LDL. In addition to this, LDL retention on these walls triggers an immune response, resulting in a cascade of production of oxidants and inflammation [142,143]. Plasma LDL interacts with circulating MPO, which has been reported to be higher in patients suffering from atherosclerosis [144]. It has been reported that in some patients undergoing hemodialysis, HOCl reacts with LDL, which promotes atherogenesis [91,145]. Macrophage exposure to HOCl^−^-LDL results in an accumulation of cholesterol and its esters as well as the production of lipid-rich foam cells [146]. 

### 3.4. Obesity

The infiltration of neutrophils in adipose tissue is an initial and persistent step for the advancement of diet-induced obesity [84,147]. This infiltration of neutrophils leads to increased MPO expression, as recent reports have also revealed a higher level of MPO in obese adults [148,149,150]. Thus, with this concern of obesity, prolonged low-grade inflammation combined with cardiovascular risk factors probably occur in parallel. A proper mechanism to justify the regulation of MPO activity in cases of obesity requires further research [151,152].

To investigate the possible role of MPO in obesity, Wang et al. [148], during his research on animal models, found that MPO-deficient mice showed resistance to progression towards diet-induced obesity. In another study, isolated neutrophils were treated with an inhibitor against MPO action, and this prevented insulin resistance in obese mice. All of these observations indicate the probability that MPO targets white and brown fat and is a chief contributor to the progression of inflammation-induced obesity, but this assumption also requires further investigation [148].

In humans, a strong positive correlation has been found between obesity and diabetes with leukocytosis [153,154,155], as the plasma MPO level was found to be raised in prepubertal obese children [85]. A study of MPO as an early biomarker of inflammation and obesity in prepubertal obese children indicated an MPO level of about 22 µg/L, as compared to normal weight children with an MPO level of about 14 µg/L. Thus, these studies support the hypothesis that serum myeloperoxidase is a suitable and potential biomarker for assessing various cardiovascular risks among obese patients [156]. 

### 3.5. Neurodegenerative Diseases

Oxidative stress is also proposed to be responsible for the release of neurotoxic mediators commanded by MPO derived from cells like neurons, astrocytes, and activated microglia, as well as peripheral inflammatory cells [11]. In the brain, of the different neurotoxic oxidants, HOCl is a stable, highly reactive, and predominant one. This acid is involved in a number of neurodegenerative diseases, including stroke, epilepsy, multiple sclerosis, Parkinson’s and Alzheimer’s diseases, and multiple sclerosis, etc. [157]. 

In addition to inflammation and oxidative stress, MPO is related to depression, which is an emotional disorder affecting a vast majority of the world’s population [158]. Furthermore, patients suffering from bipolar disorders are prescribed anti-inflammatory drugs, such as lithium and valproate, indicating some links with MPO [159]. Still, the complete mechanism of the role of myeloperoxidase biochemistry in neuronal diseases remains unknown.

### 3.6. Diabetes/Diabetic Retinopathy

Diabetes mellitus and its complication, diabetic retinopathy (DR), are also known as diseases with subclinical inflammation. During diabetic retinopathy, retinal structural and biochemical alterations cause the activation of neutrophils [160]. The increased expression of various types of growth factors and cytokines including TNF-α occurs due to biochemical modifications during DR. Inflammatory mediator priming causes MPO translocation and interaction with anti-MPO antibodies. 

In the vessels of diabetic retina, the upregulation of leukocyte adhesion molecules occurs, and leukocytes are also observed in the lumen of human microaneurysm. Furthermore, the vitreous samples of patients with DR show elevated levels of CD4/CD8 and T lymphocytes [90]. 

Chronic inflammation during diabetic retinopathy is sustained by cytokine-producing B-lymphocytes. There is a correlation between the activity of proliferative DR (PDR) and increased lymphocyte infiltration [161]. Increased vascular permeability due to leukocytosis leads to retinal abnormalities, endothelial injury, and capillary occlusion [162,163,164]. Neutrophils and monocytes can be activated by proteinase-3 antineutrophilic cytoplasmic antibody (PR3-ANCA) and MPO antinutrophilic cytoplasmic antibody (MPO-ANCA) to release acute inflammatory mediators, which causes endothelial cell injuries [165,166]. Priming by proinflammatory factors, such as cytokines, TNF-α, and microbial products like bacterial formyl peptides, etc., triggers circulating neutrophils to express more ANCA antigens. 

### 3.7. Liver Diseases

Among several types of liver disease, such as fibrosis, necrosis, inflammation, and steatosis, alcoholic cirrhosis denotes a major cause of mortality with an estimated 3.8% of all worldwide deaths [167,168]. Cirrhosis is closely related to immune dysfunctions, and thus to the inability of the host to protect against various infections [169]. In several types of liver injuries, for example alcoholic steatohepatitis in human beings or in animal models, neutrophils contribute to the pathogenesis of cirrhosis [170]. The infiltration of neutrophils in the liver is good for predicting disease [93], as these cells increase the intracellular oxidative stress during liver injury [171]. In addition to this, the stellate macrophages or Kupffer cells located in the liver also synthesize MPO. The activation of these cells results in hepatic fibrosis, which is proposed to be developed by the local release of oxidants and cytokines [92,172]. 

Neutrophils employ their favorable effects through different factors such as granulopoiesis [144,173], the production of hepatocyte growth factor [174], and collagen degradation. Granulocyte-colony stimulating factor (G-CSF) therapy has been observed to be beneficial in cases with severe alcoholic hepatitis [175]. Patients with cirrhosis have impaired neutrophilic ROS production, as well as phagocytotic and microbicidal activities [176,177,178]. Post-hepatic cirrhosis has also been observed to be closely related to diminished ROS production in some liver transplant recipients [179]. The mechanism of impaired signaling events of neutrophils in relation to alcoholic cirrhosis is not fully understood. Several researchers have observed an erroneous MAPK-dependent phosphorylation of p47phox, an important component of NADPH oxidase [30].

### 3.8. Cancer

The knowledge of the precise biochemical relationship between the inflammatory response and specific malignancy is a vast field to be understood, although growing evidence points to links between the relationships of MPO, inflammation, and cancer [88,180,181]. Cancer progression advances by the biochemical alterations of different biomolecules and genes by various oxidative species, ultimately produced through MPO.

DNA damage can be caused by oxidants directly or indirectly produced by MPO, which can lead to mutagenesis [86]. An abnormal MPO expression and greater risks of different forms of cancers are directly associated with MPO gene polymorphism [182]. In the promoter region of this peroxidase gene, single nucleotide polymorphisms (SNPs) can possibly affect transcription and protein levels [87,183]. In addition to this, the substitution of thymidine for cytosine in codon 569 causes the substitution of an amino acid from arginine to tryptophan, which may also cause some genetic defects of MPO [184]. 

In addition to gene polymorphism, MPO induces cancer through the activation of genotoxic intermediates and procarcinogens through an indirect implication of MPO [185,186]. The metabolism of unsaturated fats and some amino acids like serine and threonine can form byproducts, like acrolein, which in turn form acrolein-protein adducts [187]. In humans, these new protein adducts can transform colon tumors from benign to malignant states [188]. Still, little information is available about such proteins that form adducts with acrolein or their role in tumor progression.

Several reports are available about the relationship between breast cancer and increased serum MPO level as compared to control groups. The promotion of this cancer is also enhanced by inflammatory leukocytes, which produce ROS, chemo- and cytokines, proteases, histamine, and other mediators [189]. Various types of DNA damages and genomic instability are instigated by MPO-synthesized ROS [180,190]. Thus, in premenopausal women suffering from breast cancer, MPO acts as an efficient marker [191]. Furthermore, risks of the development of cancer are directly linked to the endogenous production of high MPO levels [192].

Myeloperoxidase is a hallmark enzyme of acute myeloid lineage and the clinical relevance of the circulating MPO level in acute myeloid leukemia (AML) patients showed higher plasma MPO levels (range 1.0–9514 ng/mL) as compared to control subjects (range 3.5–20.6 ng/mL) [193].

### 3.9. Cystic Fibrosis

Cystic fibrosis (CF), a disease of the respiratory tract, is characterized by severe bacterial infections, especially *Pseudomonas aeruginosa*, as well as very large numbers of infiltrating neutrophils [194]. Neutrophils are also thought to contribute to lung damage instead of eliminating bacteria from the respiratory tract [94]. Cystic fibrosis patients’ sputum contains high concentrations of MPO and human neutrophil elastase (HNE), and these levels correlate with the severity of the lung disease [195,196]. The clear mechanism for the release of inflammatory mediators like HNE, extracellular DNA, and MPO from neutrophils during CF is not known. However, neutrophil extracellular traps reveal a potential mechanism for the release of these mediators [197]. 

## 4. Myeloperoxidase Deficiency

Several studies have shown that, in the USA and Europe, partial or complete MPO deficiency is relatively common among the human population (affecting 1 in 2000 to 1 in 4000 people) [198,199]. However, there is a geographic heterogeneity between the frequencies of hereditary MPO deficiency in different populations. For example, these findings are compared to the reported 1 in 55,000 in Japan [200,201,202]. Generally, MPO deficiency results in a modest increase of either inflammatory problems or infectious complications [203]. MPO-deficient neutrophils exhibit impaired bactericidal and candidacidal activities against *Staphylococcus aureus* and many species of *Candida* [204,205]. 

The deficiency of MPO is a hereditary problem that may also lead to immune deficiency [206]. In addition, several different types of autoantibodies have been observed to be raised against MPO in various types of vasculitis. The three most clinically prominent vasculitis forms of this type are granulomatosis with polyangitis, and eosinophilic granulomatosis with polyangitis (EGPA) and microscopic polyangitis. Autoantibodies against neutrophils, also known as ANCAs, have also been detected in the perinuclear region staining [207]. 

## 5. Conclusions

Myeloperoxidase enzyme is the most abundant pro-inflammatory biomarker present in neutrophilic granulocytes. It is released from these cells by proinflammatory factors and during oxidative stress at the site of infection to combat different types of microbial activities. The antibacterial activities of MPO involve the production of different reactive oxygen and nitrogen species. Myeloperoxidase also plays a role in the chemical modifications of different lipoproteins, protein nitrosylation, tyrosyl radical formation, and dityrosine crosslinking, etc. Myeloperoxidase gained special importance as a well-known biomarker due to its role in a number of inflammatory diseases including rheumatoid arthritis, cardiovascular diseases, neurodegenerative diseases, diabetic retinopathy, liver diseases, cancer, and transplant rejection. As the activation of macrophages and neutrophils may occur in any type of inflammation, significant future research is required to precisely understand the role of MPO in these diseases. Different assays have been conducted to check the level of MPO in several diseases due to the lack of a specific substrate, but no comparisons have yet been made between these assays. Thus, it is very important to standardize the assays of MPO with some specific substrate with the aim of understanding the reference range of MPO in different diseases.

## Figures and Tables

**Table 1:** Brief etiology and the direct/indirect involvement of MPO in different types of diseases.

No.	Name of Disease	Brief Etiology and Possible Role of MPO	Reference
1	CVD and atherosclerosis	Raised level of MPO causes RBCs deformability, accumulation of cholesterol and its esters, ruptures in atherosclerotic plaque	[8,60]
2	Obesity	Neutrophil infiltration and activation of MPO in adipose tissue	[84,85]
3	Neurodegenerative diseases	Release of neurotoxic mediators by many factors spearheaded by MPO from neurons, astrocytes, microglia cells	[11]
4	Cancer	MPO-derived ROS/RNS react with major biomolecules causing mutagenesis, gene polymorphism, SNPs, acrolein-protein adduct formation	[86,87,88]
5	Diabetes/diabetic retinopathy	Neutrophil activation and the release of MPO in vessels and retina, upregulation of leukocyte adhesion molecules, and increased production of anti-MPO antibodies	[89,90]
6	Renal diseases	MPO-initiated HOCl-modified proteins in glomerular peripheral basement membranes	[91]
7	Liver diseases	Neutrophil infiltration, hepatic fibrosis by activation of Kupffer cells cause production of oxidants, impaired signaling events	[92,93]
8	Lung injury	Activation and expression of proinflammatory cytokines and mediators by MPO	[5]
9	Cystic fibrosis	Bacterial infiltration, especially *Pseudomonas aeruginosa* and infiltrating neutrophils	[94]
10	Multiple sclerosis	MPO-generated ROS cause axonal damage by proteolytic enzymes and cytotoxic oxidants by activated immune cells and glia	[95]
11	Alzheimer’s disease	Increased production of oxidants like advanced glycation end products, *o*,*o*′-dityrosine, lipid oxidation products, protein carbonyls, oxidized DNA, and 3-nitrotyrosine in neuronal tissues proposed by increased expression of MPO	[96]
12	Parkinson’s disease	Upregulation of MPO and its byproduct, 3-chlorotyrosine, in ventral midbrain	[97]
13	Tuberculosis	Enhanced MPO expression along with TNF-α and IL-12 activation	[98]
14	Asthma	Excessive MPO release from neutrophils in lower respiratory tract cells	[99]
15	Rheumatoid arthritis	Inflamed synovium intervened by lymphocytes and neutrophils leads to the release of proinflammatory mediators	[100,101]
16	Chronic sinusitis	Enhanced level of MPO and IL-8 in sinuses	[102]
17	Peptic ulcer	Free radicals formation initiated by MPO	[103]
18	Gastric ulcer	Neutrophil infiltration and the release of MPO into gastric mucosal tissue	[104]
19	Duodenal ulcer	MPO and other pro-inflammatory agents	[105]
20	Colitis	Increased activity of MPO and pro-inflammatory mediators like IL-1β and TNF-α	[106,107,108]
21	Pancreatitis	Increased MPO activity causes increased ROS that leads to this disease	[109]
22	Chronic periodontitis	Increased MPO activity in gingival crevicular fluid	[110]

MPO: myeloperoxidase; CVD: cardiovascular disease; RBCs: red blood cells; ROS: reactive oxygen species; RNS: reactive nitrogen species; SNP: single nucleotide polymorphism; IL: interleukin; TNF-α: tumor necrosis factor-α. The descriptions of some of the diseases through the perspective of MPO are reviewed in this paper.
